# Bias Characterization in Probabilistic Genotype Data and Improved Signal Detection with Multiple Imputation

**DOI:** 10.1371/journal.pgen.1006091

**Published:** 2016-06-16

**Authors:** Cameron Palmer, Itsik Pe’er

**Affiliations:** 1 Department of Systems Biology, Columbia University Medical Center, New York, New York, United States of America; 2 Department of Computer Science, Columbia University, New York, New York, United States of America; University of Washington, UNITED STATES

## Abstract

Missing data are an unavoidable component of modern statistical genetics. Different array or sequencing technologies cover different single nucleotide polymorphisms (SNPs), leading to a complicated mosaic pattern of missingness where both individual genotypes and entire SNPs are sporadically absent. Such missing data patterns cannot be ignored without introducing bias, yet cannot be inferred exclusively from nonmissing data. In genome-wide association studies, the accepted solution to missingness is to impute missing data using external reference haplotypes. The resulting probabilistic genotypes may be analyzed in the place of genotype calls. A general-purpose paradigm, called Multiple Imputation (MI), is known to model uncertainty in many contexts, yet it is not widely used in association studies. Here, we undertake a systematic evaluation of existing imputed data analysis methods and MI. We characterize biases related to uncertainty in association studies, and find that bias is introduced both at the imputation level, when imputation algorithms generate inconsistent genotype probabilities, and at the association level, when analysis methods inadequately model genotype uncertainty. We find that MI performs at least as well as existing methods or in some cases much better, and provides a straightforward paradigm for adapting existing genotype association methods to uncertain data.

## Author Summary

Genetic research has been focused at analysis of datapoints that are assumed to be deterministically known. However, the majority of current, high throughput data is only probabilistically known, and proper methods for handing such uncertain genotypes are limited. Here, we build on existing theory from the field of statistics to introduce a general framework for handling probabilistic genotype data obtained through genotype imputation. This framework, called Multiple Imputation, matches or improves upon existing methods for handling uncertainty in basic analysis of genetic association. As opposed to such methods, our work furthermore extends to more advanced analysis, such as mixed-effects models, with no additional complication. Importantly, it generates posterior probabilities of association that are intrinsically weighted by the certainty of the underlying data, a feature unmatched by other existing methods. Multiple Imputation is also fully compatible with meta-analysis. Finally, our analysis of probabilistic genotype data brings into focus the accuracy and unreliability of imputation’s estimated probabilities. Taken together, these results substantially increase the utility of imputed genotypes in statistical genetics, and may have strong implications for analysis of sequencing data moving forward.

## Introduction

Genome-wide association studies (GWAS) have become a primary tool to elucidate the correlations between SNP genotypes and complex phenotypes in large cohorts. Association studies initially assumed the existence of genotype calls: for each sample at each assayed variant, either reference allele homozygote, heterozygote, or alternate allele homozygote. As such, methods developed for analyzing GWAS assumed the existence of such perfect-confidence genotype data. The association study design and related analysis methods have remained in force even as the field has transitioned into the sequencing era and more complete data have become available. In all situations, due to technical and financial limitations, association studies only partially assay the set of common variants in any organism. The variants included on a SNP array typically only include a small fraction of the total pool of variants present, and even sequenced variants are called incompletely and inconsistently. Furthermore, due to the low magnitude of effect of most trait-associated variants, studies prioritize sample size via multisite meta-analysis, involving genetic samples assayed on different technologies. This study design results in a complicated missingness pattern across the entire conceptual set of common variants in a sample. Yet due to shared linkage disequilibrium between different samples, this missingness can be overcome with the addition of external reference data.

Genotype imputation probabilistically estimates unknown genotypes for a study sample by leveraging external reference haplotypes ascertained at a superset of SNPs [1]. Genotype calls and genotype probabilities are fundamentally different. Genotype calls are considered certain data and, under the traditional additive model, may be represented as an integer count of a reference allele present for each study individual. Direct extension of these simple models to probabilistic data is not possible, as the models are not capable of representing the additional variance component introduced by uncertainty. This is critical to understanding the traditional challenge in analyzing imputed genotype data: existing methods are not directly compatible with uncertain data, so probabilistic genotypes must be projected to a lower-dimensional approximation resembling genotypes, with a concomitant loss of information and introduction of bias.

Three primary methods have been developed for the handling of uncertain data in genetic association studies. For the first method, and at one extreme, probabilities may be converted to call-like integral counts by choosing the class with the largest probability. This paradigm requires no additional modification of the analysis method for genotype calls, but all meaningful information about uncertainty is lost. For the second method, in some situations, genotype probabilities may be converted into expected counts of the reference allele: the “allelic dosage.” This strategy attempts to maintain some of the uncertainty of the genotype estimate by allowing non-integral values: for example, when the heterozygote and reference allele homozygote classes are equiprobable and the alternate allele homozygote has 0 probability, a sample is considered to have 1.5 alleles. Unfortunately, this strategy is only useful when the underlying algorithm extends to nonintegral data (for example, a generalized linear model with continuous predictor); furthermore, there is no rigorous proof of the degree of bias or information loss incurred using this method. Finally, as a last method, the algorithm may be modified to directly operate on probabilities [2]. This option attempts to include all uncertainty information at the cost of additional work creating a new algorithm. In practice, this type of custom algorithm design is limited to simple GLM methods; other studies in the field creating more complex statistical models for association studies do not undertake this additional work [3–8].

In the statistics literature, Multiple Imputation (MI; distinct from genotype imputation; [9]) is the rigorous method of conducting analysis on probabilistic estimates of uncertain data. The details of MI are discussed in Methods. Briefly, a small number of complete datasets are randomly drawn according to the probability data. These datasets are then analyzed using any standard analysis technique that generates a normally-distributed effect estimate *β* and standard error estimate *s*. Importantly, MI is not a method of estimating hidden data, but rather a method of handling existing estimates. The performance of MI, and indeed of all imputed data analysis methods, is reliant on the quality of the underlying genotype imputation. Imputation accuracy, the agreement between predicted and true genotype, tends to vary across both imputation “quality,” as estimated by most imputation software, and minor allele frequency. The extent of this variable performance has not, to our knowledge, been rigorously assessed.

In this study, we seek to rigorously evaluate this variable genotype imputation performance. We show a significant deviation between genotype probabilities generated by imputation, and empirical probabilities estimated at the same sites. This failure of probability consistency is an important confounding effect in imputed data analysis. We show that Multiple Imputation matches or improves upon performance of existing imputed data analysis regimes by better prioritizing true positive associations, while additionally being straightforwardly extensible to future analysis algorithms.

## Results

### Imputation Accuracy

**Fig 1:**
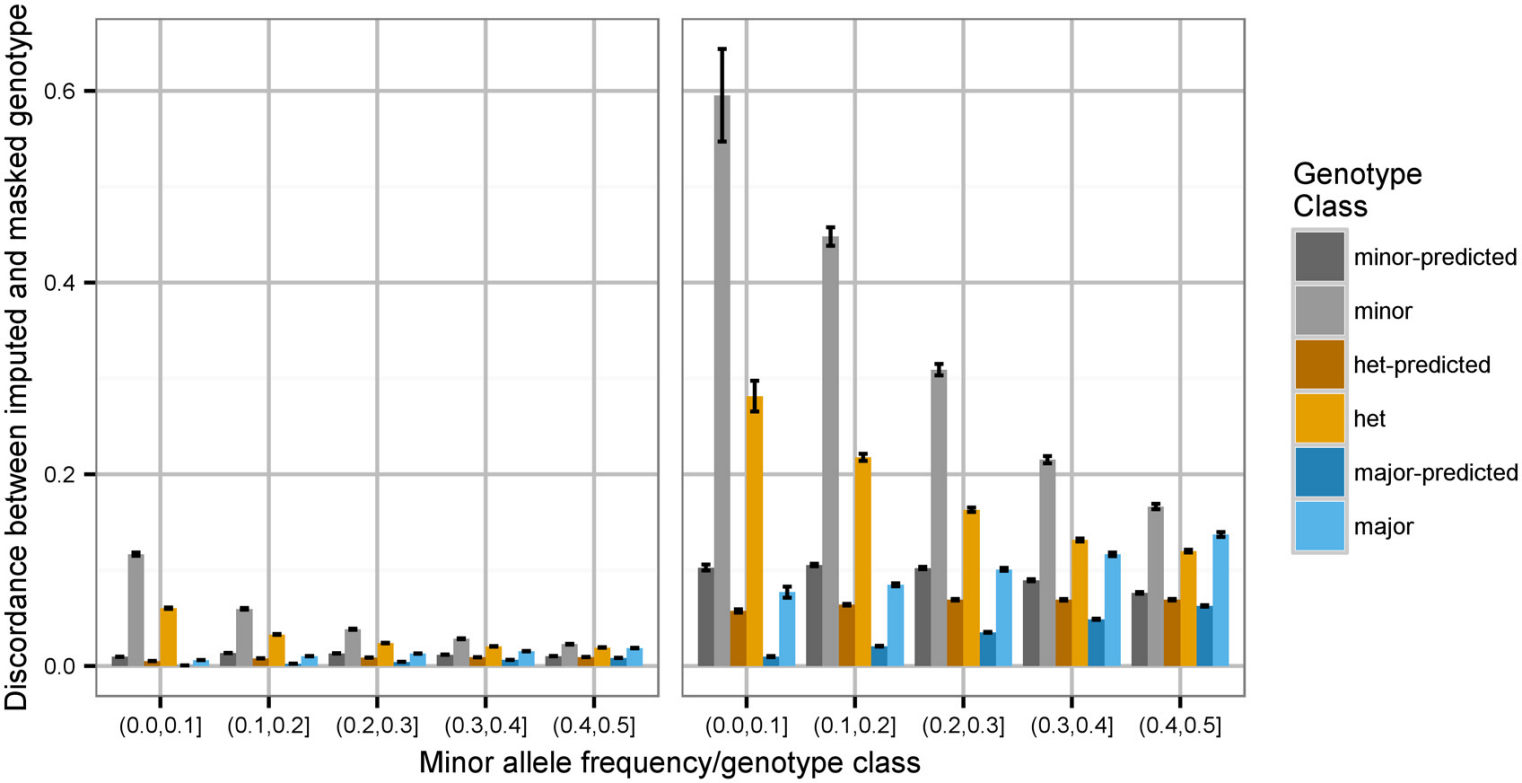
Relationship between quality of estimated allelic dosage from IMPUTE2 imputation and predictors of imputation quality. Data are estimated from 10% of the original chip (59808 SNPs) masked from imputation. Discordance of predicted allelic dosage (y axis) is the fraction difference between dosage computed from imputation probabilities and dosage based on masked genotype data: for example, if the true genotype is reference homozygote and the allelic dosage from imputation is 1.4, the discordance is $\frac{\left|2-1.4\right|}{2}=0.3$. Left panel: imputation quality greater than 0.9; right panel: quality between 0.8 and 0.9. Clusters correspond to minor allele frequencies of 10%; individual bars represent quality stratified by masked genotype. “Predicted” bars correspond to expected concordance assuming independence of individual haplotypes. Error bars represent 95% confidence intervals of mean discordance estimate.

We compared the allelic dosage (Methods) to the “true” genotype count based on masked genotype data. Fig 1 shows the fraction error between allelic dosage from imputation and masked genotype, stratified by genotype class, allele frequency, and reported imputation quality metric (hereafter called “*r*^2^”). We observe that a single quality metric *r*^2^ masks significant deviations in mean quality between different genotype classes and different allele frequencies. For imputed SNPs reported to be of high quality (*r*^2^ > 0.9, left panel; top 40% of GWAS-used SNPs, S1 and S2 Figs), and with sufficiently high minor allele frequency, imputation is indeed well-behaved: variants with minor allele frequency above 0.3 have less than 3% discordance with similar performance across genotype classes. However, SNPs of lesser imputation quality (0.8 ≤ *r*^2^ ≤ 0.9, right panel; approximately 18% of GWAS-used SNPs, S1 and S2 Figs) or low minor allele frequency are inconsistently imputed. Minor allele homozygotes and heterozygotes in particular are subject to highly inflated error rates. While differences across the SNP strata are observed with both IMPUTE2 and minimac3 imputations, the magnitudes observed are distinct (S3 Fig). Equivalent results are observed when evaluating performance by fraction of best-guess genotypes from imputation not matching masked genotypes (S4 Fig).

### Imputation Probability Consistency

**Fig 2:**
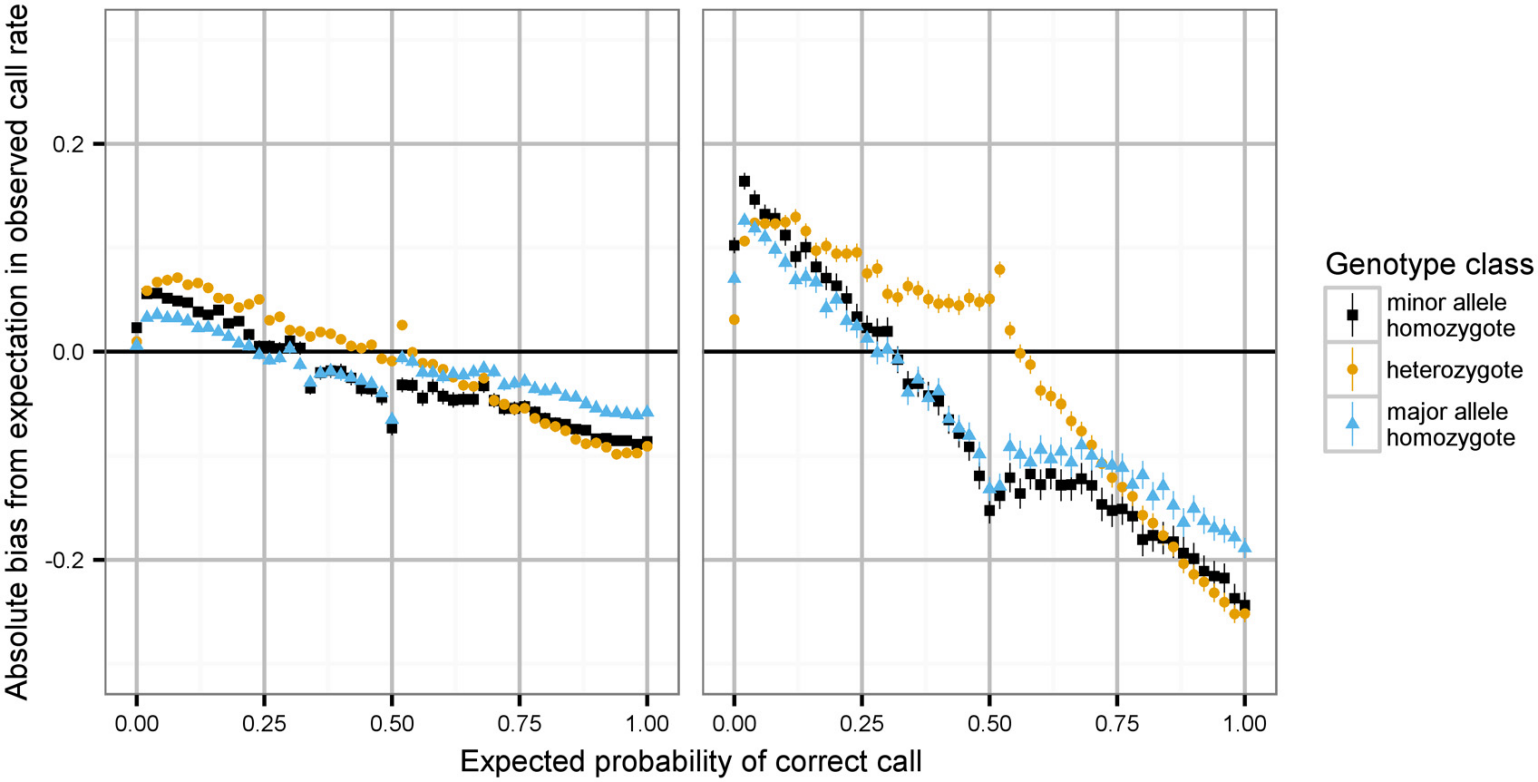
Evaluation of the consistency of probability scores from IMPUTE2 imputation. Data are estimated from 10% of the original chip (59808 SNPs) masked from imputation. x-axis: 0.02-width bins of imputation probabilities; y-axis: mean deviation between expected and observed accuracy. Data series correspond to results stratified by genotype class. Left panel: IMPUTE2 info metric greater than 0.9; right panel: info metric between 0.8 and 0.9. Error bars represent 95% confidence intervals around mean consistency estimate.

We next examined the imputation probabilities themselves, to evaluate whether probabilities generated by imputation software correspond to the empirical probability of observing a genotype at a particular site. Results for this comparison for IMPUTE2 probabilities are shown in Fig 2, across strata of reported quality and predicted call probability. The empirical accuracy significantly deviates from the predicted, and much more so with decreasing *r*^2^; S5 Fig shows similar plots comparing the effect of decreasing minor allele frequency on this distortion, and show weaker but significant changes with decreasing frequency. Of note, the heterozygote genotype class behaves in a distinct but complementary fashion relative to the two homozygote classes.

These results are distinguishable between imputation programs: the effect is much stronger in the IMPUTE2 imputation. The most substantial difference between the two programs is IMPUTE2’s use of sequential imputation windows to improve performance through parallelization, with potential accuracy tradeoffs, yet we have observed no differences caused by modifying this parameter. We note that differences in imputation performance under different conditions have been observed extensively and unpredictably (see, among many, [10–14]). Our observations are consistent with intermittent observations of MACH-family algorithms nominally outperforming IMPUTE-family algorithms in some cases, yet the precise reason(s) for these differences between similar software has never, to our knowledge, been demonstrated.

### Signal Enrichment with Multiple Imputation

We next sought to evaluate whether the ability to prioritize verified trait-associated SNPs in an association study ranking was detectably different using MI or other existing algorithms. We considered 73 replicated loci from a large (N = 339224 individuals) GWAS for BMI [15]. As expected with our modest sample size of 2802, we have little power to detect the majority of these variants at genome-wide significance 5 ⋅ 10^−8^. Nevertheless, if the variants are associated with the trait at all, one expects the variants to be relatively better ranked in the final list of variants than variants chosen at random from the study. We conducted a BMI genome-wide association study in Health ABC, as discussed in Methods. Using these SNP association results, we computed the rank percentile of each published variant, comparing these results for two existing imputed data analysis algorithms and MI.

**Fig 3:**
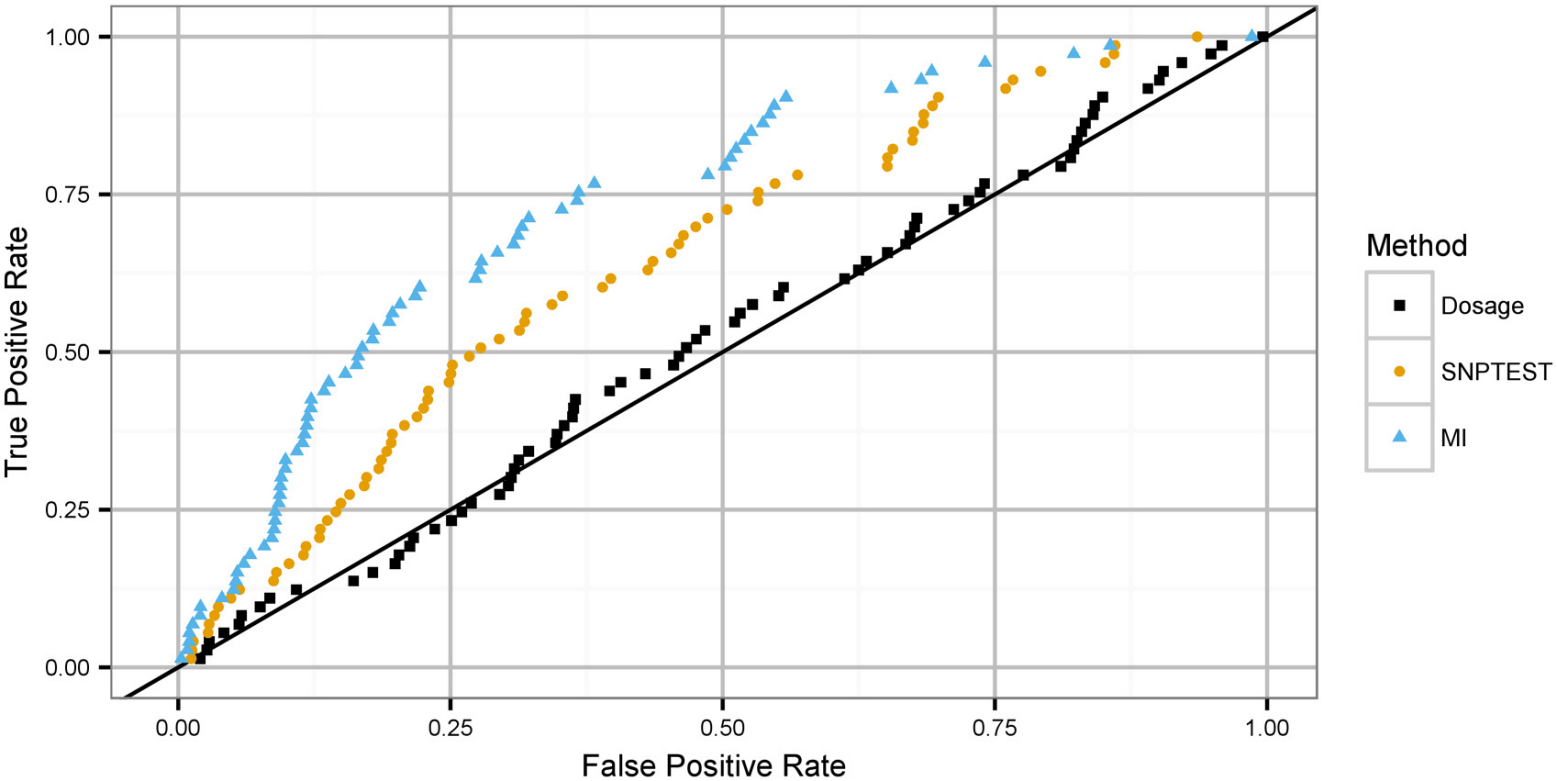
Receiver operating characteristic curves for Health ABC BMI association with IMPUTE2 imputation. True positive associations are 73 established BMI variants from [15]. Tracks correspond to uncertainty handling methods: allelic dosage, score test, MI on imputed probabilities.

We evaluated the receiver operating characteristic curves for PLINK dosage, SNPTEST score, and MI (Fig 3). MI significantly outperforms all other methods (one-tailed DeLong test *p* < 2 ⋅ 10^−16^). We repeat this analysis with a height GWAS in NFBC66 (Methods), and find a similar significant improvement in signal detection by MI compared to other methods (one-tailed DeLong test *p* < 0.0003764). We conclude from this analysis that significant information loss may occur when uncertainty is incompletely handled in imputed data analysis. There is no evidence in this comparison that Multiple Imputation is inferior to other methods.

### Changes in Null Distribution of Variants

Improved signal detection by MI may be attributed to several causes. In observing the additional variance component in the multiple imputation model, $s_{B}^{2}$ (see Methods), we note that variability introduced by genotypic uncertainty should result in decreased rankings for variants regardless of trait being analyzed. Uncertainty in probabilities also differs with variant-wide imputation quality *r*^2^, implying that different association tests may lead to different expected distributions of variants in a ranked association study across the quality spectrum, whether variants are trait-associated or not.

**Fig 4:**
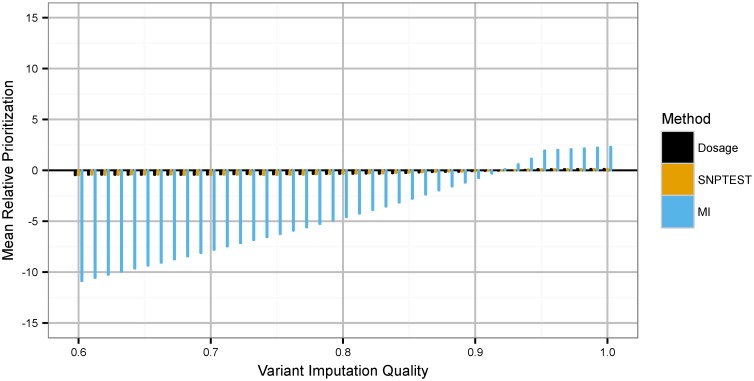
Evaluation of the null distribution of variants across an association study ranking. x-axis: variant imputation quality bin; y-axis: absolute deviation from uniform random expected rank across all variants at given quality (that is, 50%). Data tracks correspond to PLINK dosage analysis, SNPTEST exact testing, and MI.

We sought to evaluate the null distribution of variant ranks across imputation quality. We calculated the percentile rank of each variant in the BMI GWAS and sorted the variants into bins across imputation quality. Results from this investigation are shown in Fig 4. All tested methods have a statistically significant correlation between imputation quality and average rank (all Pearson correlation test *p* < 2 ⋅ 10^−16^; comparable results for nonparametric tests). PLINK and SNPTEST have indistinguishable magnitudes of effect (shared effect size -0.017, indistinguishable with *p* = 0.44). Multiple Imputation shows a significantly stronger correlation than the other tests (effect size -0.351, greater than other tests with *p* = 3.4 ⋅ 10^−7^). MI produces test statistics that much more significantly incorporate uncertainty, through in particular the between-draw variance component.

Published trait-associated variants tend to impute with higher quality than random variants from a dataset due to selection bias, leading to the possibility that this global incorporation of quality by MI is leading to better performance in a manner unrelated to the trait itself. To control for trait-unrelated differences between published variants and the global SNP distribution, we computed empirical matched null sets for every published variant by drawing 1000 SNPs matched to this variant with parameters *r*^2^ ± 0.01 and frequency ±0.01 of the true variant. Here we are drawing variants from the association study itself; this null allows us to detect effects specifically affecting the significant tail of the distribution over the bulk of variants. For most traits, these variants will overwhelmingly be unassociated with the outcome, and thus this null will correspond to a true null of no genetic association; in the case that this condition is untrue, this null will lead to relatively conservative assumptions about the added performance of MI. We then regenerated ROC curves for each analysis, this time controlling for the null rank of SNPs matched to the published variant list based on these parameters. These results approximately correspond to trait-specific enrichment effects caused by the various analysis methods. We find that adjustment for trait-secular shifts in variant quality affects BMI and height differently. For BMI, MI continues to outperform PLINK and SNPTEST (one-tailed DeLong *p* < 1.177 ⋅ 10^−10^). For height, null adjustment reverses the previously-observed trends, such that MI tends to underperform PLINK and SNPTEST (one-tailed DeLong *p* < 0.0025). We note that for this trait in particular, the null derived from drawing from the association study itself may be conservative given the broad genetic basis for human height [16].

### Extensibility

To underscore the benefits of MI not simply on regression with existing uncertainty handling methoods, but additionally on more complex algorithms more difficult to directly adapt, we applied MI to EMMAX [4]. We ran EMMAX on both the Health ABC BMI GWAS and the NFBC66 height GWAS, with default parameter settings and the IBS-based kinship matrices. We compared EMMAX with thresholded genotype data and MI on imputed probabilities. The published version of EMMAX only accepts integral count genotypes, thus no other comparisons were included in this test.

We detect significant improvement in relative percentile increase for BMI-associated variants that is abrograted by null adjustment (one-sided DeLong test *p* = 6.663 ⋅ 10^−12^ and *p* = 0.09933 respectively). We find weak evidence for improved overall efficiency for known height variants (one-sided DeLong test *p* = 0.01994), which under null adjustment becomes a significant underperformance of MI relative to rounded genotype control (one-sided DeLong test *p* = 2.242 ⋅ 10^−7^); again, we note that this null may be overly conservative in this trait context [16]. Evaluation of the null distribution of variants for this experiment, analogous to the analysis in Fig 4, shows that the MI-mediated rank shift is much smaller across both the frequency and variant quality spectrum. We propose that the error model used in EMMAX is partially compensating for the additional uncertainty variance component redundantly modeled by MI.

## Discussion

In this paper, we analyze the application of Multiple Imputation in the particular context of genotype imputation. We find that representative existing analysis methods tend to increase noise in association testing by incompletely modeling the uncertainty of genotype estimates across the entire set of imputed variants. This results not just in lower quality variants, for which information is limited, receiving inappropriately high ranking, but variants for which underpowered but significant trait association is present becoming lost in statistical noise introduced by imputation. We furthermore detect imputation program-specific inconsistencies in posterior genotype probabilities that differently affect the three genotype classes. Overall, we characterize the complexity of imputation probabilities and show that MI can improve association testing with uncertain data at the cost of increased post-imputation computational time.

This study particularly elaborates on the interpretation of a *p*-value from a probabilistic association study. Based on the results in Fig 4, we see that existing analysis tends to rank variants uniformly across *r*^2^: the *p*-value weakly incorporates imputation quality, enough so that the tests are not correctly calibrated but insufficiently so to actually correct for differential uncertainty. This poor calibration is verifiable with a true null: observing the null distribution of variants in our study regressed against random standard normal traits, we observe a comparable correlation between variant imputation quality and SNP ranking for SNPTEST (p = 0.001953). Dosage analysis does not, in that context, recapitulate the correlation effect observed in our data, suggesting that the trend is specific to our trait-associated null. This poor calibration is potentially shared by many analysis methods in the field: following the simulation work of Acar and Sun [17], we have found low magnitude but significant miscalibration when operating on probabilistic genetic data and null traits for linear regression and a Kruskal-Wallis test on best-guess variants; dosage analysis; and their generalized Kruskal-Wallis test that directly handles uncertainty (all Wilcoxon rank sum *p* < 2 ⋅ 10^−16^). There is also a significant correlation between uncertainty for the Kruskal-Wallis based tests in this simulation context (ANOVA *p* < 2 ⋅ 10^−16^). Association p-values are intrinsically affected by genotype uncertainty, altering the expected null distribution from uniform random, but this is simply an uncharacterized bias introduced by incompletely handling uncertainty, not a formal characteristic with proven properties.

We suggest that this behavior is undesirable: if a SNP estimate is uncertain, then the behavior of an ideal inference should be to proportionally downweight the ranking of that SNP in that particular study in a predictable fashion. With Multiple Imputation, this uncertainty-induced variability is correctly apportioned to the between-draw variance component $s_{B}^{2}$, resulting in unmodified effect estimates that are still suitable for cross-cohort meta-analysis. In the case of our example BMI association study, the positive control variants used were all imputed with high confidence, yet MI still showed a trait-specific rank improvement in these SNPs, due to the selective downranking of low confidence variants with no or lesser phenotype association. In short, we see little reason to continue using dosage or score test methods for handling imputation probabilities, when a straightforward alternative with superior statistical properties exists.

The results in Figs 1 and 2 broadly characterize imputation performance across variant quality, frequency, and genotype class. Although parts of these results are hinted at in various imputation papers (in particular, [18], but also [2, 12, 19, 20]), we have not before encountered a presentation of the severity of quality degradation across the full spectrum of frequencies. We urge analysts in all contexts to avoid genotype thresholding, in particular using IMPUTE2 probabilities: with decreasing frequency and quality, the likelihood of rounding to an incorrect genotype grows extremely high. We also observe strong per-genotype class differences in performance, most severly affecting heterozygotes. This is not a surprising result, given the integral nature of computational prephasing in genotype imputation. In the modern genotype imputation protocol, prephasing is a one-time burdensome computation that is not repeated, whereas the imputation step itself is comparatively rapid and repeated many times as new reference panels become available. A useful future analysis would investigate the specific impact of differential phasing quality on imputation probability bias, and our results emphasize that prephasing should be reconducted as advancements in the field yield higher quality phasing solutions.

We have observed in the literature a tendency to ignore uncertainty in genotype data when developing new algorithms. This elision is understandable in the sense that complete-case analysis is typically substantially more straightforward to implement, and as a first approximation, uncertain data may be converted seemingly straightforwardly to genotype “calls” via techniques such as rounding. Yet in the case study of simple regression, we see that this approximation bears with it a cost in loss of statistical power to detect trait-associated variants. One of the great benefits of MI is its simple application to complete-case methods, as each round of MI generates complete case data. In the case of standard regression, this is a straightforward benefit; in the case of a method such as EMMAX, one must balance the desirable avoidance of rounded probabilities with potential complications of the standard MI variance component model.

One of the strongest justifications for the widespread use of imputation is the facilitation of multisite multicohort meta-analysis, in which summary statistics from separate association studies are combined to increase statistical power. The benefits of integrating imputation quality into inference are magnified in this context. Under the current regime, either {*β*, *s*^2^} or {*N*, *p*} pairs are the exclusive data provided to meta-analysis tools [21], leading to a downstream analysis that treats different estimates of variants with different imputation qualities as estimates identical to one another in expectation ascertained with perfect confidence. With nested model multiple imputation ([22], Methods), the improved model described in this paper may be extended to meta-analysis. A nested model MI meta-analysis will explicitly compensate for variable imputation quality across contributing studies with inflated variance components corresponding to noise in the mean effect estimate or inflated standard error from contributing studies (i.e., imprecision in effect estimate introduced either by variable imputation quality, fluctuations in allele frequency, or differential effect due to LD changes in different studies). The cost of this extended meta-analysis regime is limited. In addition to the standard {*β*, *s*^2^} pairs currently used to combine analysis, the within- and between-draw variance components must also be submitted. These data require additional disk space but are relatively trivial to provide. Crucially, the summary statistics from each individual draw are *not* required for this meta-analysis, such that the growth in memory requirements for meta-analysis does not scale with the number of draws conducted.

We note that by explicitly handling variable imputation quality in different studies, this meta-analysis regime introduces potential sources of heterogeneity in the final meta-analysis result. Studies with aberrantly high uncertainty may strongly influence the resulting meta-analyzed association statistic. Note that this source of heterogeneity already exists, but is not rigorously modeled and currently must be addressed by *ad hoc* filtering and quality control. We propose that heterogeneity from differential imputation quality may be quantified by a heterogeneity test analogous to the effect estimate heterogeneity *I*^2^ metric in METAL. This test would quantify heterogeneity specifically in the between-test variance component $s_{B}^{2}$ from contributing studies, which captures the noise between individual MI draws introduced by meaningfully uncertain data. This test would enable standardized detection of cases in which low imputation quality may require custom secondary genotyping for validation.

In the statistics literature, starting with [9] and moving forward, the recommended number of MI draws has varied widely. The original recommendation was that oftentimes 3–5 draws were sufficient to retain most of the accuracy while minimizing computational burden. More recent publications (i.e. [23]) have suggested that the original estimates of draws were insufficiently stringent. In this paper, we have used an *a priori* setting of 10 draws after testing various draw numbers’ effects on quality of MI output, and balancing this impact with the added burden of multiple additional MI rounds. We find that this draw count surpasses the point at which MI effect estimates tend to converge (S6 Fig), but may in the case of particularly poorly imputed variants lead to suboptimal estimates of between-draw variance (S7 Fig). A useful target for future work would be the integration of dynamic computation of the number of draws for convergence for individual variants, which in this study is complicated by the manner in which our software interfaces externally, rather than reimplements internally, with existing analysis tools to maximize compatibility.

The existence of program-specific differences in the consistency of imputation probabilities is an intriguing result that raises meaningful questions for the field. Though each program has its own algorithmic features and drawbacks, the practical choice between imputation software often reduces to the banal: ease of use, runtime and memory requirements. These features are important for imputation study designs that may take weeks to months to complete. Yet here we show a more substantive trend of probability bias that follows a distribution specific to particular programs. The precise cause of these distortions is not clear. Further investigation may be warranted to determine a method of adjusting the native probabilities generated from imputation using empirical distributions based on masked comparison data.

We interpret the potential performance improvement of MI over other methods as a call to reevaluate the use of thresholded genotype calls in other contexts. In particular, as the field continues transitioning to sequence-derived variant data, the impact of thresholded certainty in sequencing data analysis cannot be overlooked. We note that standard methods for rare variant analysis, including burden testing, adapt to the high uncertainty in low frequency variant calls by using strict quality control and relying on bulk information to resist the noise introduced by false positives. Yet interpreting the probabilistic output of sequencing technology as a mechanism for completing data in a NMAR setting, Multiple Imputation straightforwardly provides a consistent solution for even complex statistical analyses.

In the case of statistical tests that analyze linked variants together, per-site marginal information is no longer sufficient, as each MI draw must be taken from the joint distribution of all tested sites. Yet with sequencing read data, this kind of analysis is not impossible: joint information can be directly estimated from shared reads, creating a computationally challenging and yet feasible method of rigorously handling genetic uncertainty without even linkage assumptions. The creation of a standard module for interfacing with sequencing read data and dynamically generating such joint probability information would be a significant contribution to the field.

Similarly, the independent draws conducted in multiple imputation implicitly assume independence of samples. In the case of pedigrees or cryptic relatedness of samples, the sampling problem becomes much more challenging. The imputation software analyzed in this study (IMPUTE2, minimac3) is designed to handle unrelated population-based samples. The marginal probabilities generated by the algorithms are not reflective of explicit joint distribution between related samples and thus are not directly compatible with correlated genotype draws without additional modeling. One could synthesize probabilities that scale with both the imputed probabilities and the Mendelian transmission rules, for example

$$P\left(\text{child}|\text{data},\text{parents}\right)=\frac{P(\text{child}|\text{data})P(\text{child}|\text{parents})}{\sum_{i=0}^{2}P\left(\text{child}=i|\text{data}\right)P\left(\text{child}=i|\text{parents}\right)}$$

Unfortunately, such an approximation of the joint distribution would not be equivalent to actually modeling the combined structure of the data in the imputation software itself: for example, when the parents and children are modeled separately, certain genotypes will be given nonzero probabilities that would be rendered impossible by Mendelian transmission. In the case of BEAGLE [11], duos and trios are specially handled according to externally specified pedigrees. The resulting genotype probabilities then are reasonably considered to be conditional probabilities: for example *P*(**child**|**data**, **parents**). In this case, the appropriate probabilistic relationships should hold: for example,

$$P\left(\text{child}=aa\right)=\left[P\left(\text{mother}=aa\right)+\frac{P(\text{mother}=Aa)}{2}\right]\cdot \left[P\left(\text{father}=aa\right)+\frac{P(\text{father}=Aa)}{2}\right]$$

and thus consistent drawing could be conducted in a trio by first drawing the child, then drawing the parent probabilities conditional on the result of the child’s draw. Ultimately, the suitability of MI to a given application is restricted to situations where the probability-generating method is itself appropriately suited.

Recent work ([24–26]) has produced algorithms (MIX, DISTMIX, ImpG-Summary) capable of imputing association statistics from summary data, without the need for individual-level genotype information. These methods offer substantial time savings relative to genotype imputation, at the cost of reduced overall quality of estimates relative to existing HMM methods. The imputed test statistic at a particular site is the mean of a multidimensional Gaussian distribution based on neighboring test statistics and linkage disequilibrium data: this intuitively corresponds to an MLE dosage estimate from genotype imputation probabilities. It is likely the method thus suffers from analogous disadvantages to those of incomplete uncertainty handling in genotype imputation. One could imagine replacing a single mean imputed test statistic with instead a set of random variates drawn from the underlying Gaussian; these draws would act as drawn genotypes in the analysis in this study. Although multiple test statistics per variant would prove less useful for analysts of individual studies, they would be more completely reflective of the uncertain nature of these estimates. Furthermore, using sample size weighting, one could generate an MI regime in multicohort meta-analysis in, for example, METAL, in which each set of drawn test statistics is used to generate a separate meta-analysis dataset, and the resulting sets are combined using MI. Such a regime would require further investigation, and most likely would require a substantial number of iterations if many contributing cohorts used uncertain input data.

The use of MI is not completely foreign to the field of statistical genetics. We note, however, that its use is very limited, and has not been extensively compared to other, prevalent methods of handling genotype probabilities. We evaluate MI in comparison to existing methods and show MI performance is typically comparable to existing methods, and in certain contexts significantly outperforms other existing algorithms. Furthermore, we emphasize the ease with which MI is extended to, conceptually, all existing and future genotype call analysis methods with little additional effort. We foresee MI as a simple and effective component of all probabilistic genotype analysis.

## Methods

### Study Datasets

For this project, we applied for access to and downloaded two SNP array and phenotype datasets from the Database of Genotypes and Phenotypes (dbGaP) [27]. The Whole Genome Association Study of Visceral Adiposity in the Health Aging and Body Composition (Health ABC) Study, dbGaP accession phs000169.v1.p1, contains 2802 individuals genotyped on the Illumina Human1M-Duo SNP array. STAMPEED: Northern Finland Birth Cohort 1966 (NFBC1966), phs000276.v2.p1, contains 5415 individuals assayed on the Illumina HumanCNV370 array. Both datasets were requested and approved under project 7955; they are available from the dedicated General Research Use collection and do not require IRB approval.

The phenotype data released under this collection are quite limited. For this study we limited analysis to simple anthropometric traits. For Health ABC, we conducted a BMI association study with BMI (*μ* = 27.4, *σ* = 4.77) determined by age (*μ* = 73.6, *σ* = 2.87) and sex (51.2% female). For NFBC66, we conducted a height association study with height in centimeters stratified by sex (*μ*_*W*_ = 153.2, *σ*_*W*_ = 68.9; *μ*_*M*_ = 286.1, *σ*_*M*_ = 66.4; 52% female); age was not included for this single-year birth cohort.

### Sample Quality Control

Datasets on dbGaP have already been subjected to a round of cleaning by their depositors. Nevertheless, for thoroughness we cleaned the SNP array data using a standard QC protocol. Briefly, variants with minor allele frequency less than 1%, Hardy-Weinberg Equilibrium *p*-value and either per-individual or per-SNP missingness greater than 5% were removed. Cryptic relatedness was estimated using genome-wide IBS estimation in PLINK [28]. A large cluster of approximate first-cousins was detected (IBS $\hat{\pi }∼0.125$). For the purposes of this analysis, whether this is indicative of unreported pedigree structure or technical artifacts in genotype collection is irrelevant, as we are not undertaking novel variant discovery, rather conducting comparisons relative to control data.

The remaining SNPs were pruned to an independent subset of SNPs using PLINK--indep with default parameters, and these variants along with the maximal independent subset of individuals were used for unrooted principal component analysis in EIGENSOFT [6]. Standard population stratification along geographical axes is observed, confounding novel variant discovery but not effecting within-sample comparisons.

To prepare for genotype imputation, SNPs with complementary variant alleles were removed from the dataset, and positions and SNP rsIDs were updated to those of the 1000 Genomes reference panel we used. Complementary allele variants are challenging to reconcile with datasets of potentially different strand alignments. This is true when handling external datasets subject to unknown prior manipulation, but in particular in the case of modern Illumina arrays, which are only annotated with challenging “TOP/BOT” annotations instead of reference strand calls.

### Prephasing and Genotype Imputation

Following modern genotype imputation guidelines [12], we first prephased our data using SHAPEITv2 [29] with the recommended parameters. Phased haplotype data were then probabilistically imputed to the Version 3 1000 Genomes Phase 1 Integrated global reference haplotypes [30] using IMPUTE2 [19]. For comparison purposes, to establish whether effects observed were specific to the software in use, in parallel we phased the genotype data using MACH [18] and imputed the resulting phased haplotypes to the same reference panel using minimac3 [20]. Using the global reference data, a large proportion of the approximately 40 million variants in the reference dataset are expected to not segregate in the study samples and impute very poorly; thus, before downstream analysis, variants with program-specific quality metric less than 0.4 were removed entirely. No per-genotype filtering was conducted, to avoid reintroducing NMAR bias.

### Standard Analysis Methods

For standard association models under generalized linear models (in this case linear regression), various methods currently exist for analyzing probabilistic genotypes. We selected two widely-used methods of analysis for the purposes of this study. The genetics software PLINK, as of version 1.07, has an “allelic dosage” method of genotype imputation, in which the additive predictor **dosage** = 2*P*(*aa*) + *P*(*Aa*) is included in a generalized linear model (note that this value is not equivalent to a perfect confidence genotype, as it is permitted to be a decimal number between 0 and 2). This method projects the two parameter posterior probability into a single dimension, thus losing information in many cases: for example, this would consider the posterior probabilities {0, 1, 0} and $\left\{\frac{1}{3},\frac{1}{3},\frac{1}{3}\right\}$ to be equivalent.

The IMPUTE2 software used for imputation in this study has an accompanying analysis software package, called SNPTEST [2]. This software comes with a custom score test for explicitly handing genotype probabilities from imputation. The software has changed substantially since initial release; the best documentation available for the probability-handling methods is at https://mathgen.stats.ox.ac.uk/genetics_software/snptest/snptest.v2.pdf. For this investigation, we solely use SNPTEST’s frequentist methods, which can explicitly handle uncertainty.

### Statistical Missingness

Genotype imputation is a discipline-specific solution to a general statistical problem called “informative missingness” [9]. Consider a conceptual data matrix *Y* containing all phenotype and covariate data for a study. Classical statistical analysis assumes *Y* is complete, containing no null entries, and that all entries are known with perfect confidence. Study designs with null entries can be compelled into this format by removing all null datapoints before statistics are performed, resulting in a so-called “complete-case analysis.”

The effect of deviations from these assumptions vary depending on the characteristics of the missingness itself. Now assume that *Y* is the conceptual matrix containing all true datapoints with perfect confidence. Missingness observed in realistic studies is encapsulated by a second matrix, *M*, where each entry *M*_*ij*_ is 1 if *Y*_*ij*_ is missing in the true study, and 0 otherwise.

Using this framework, missingness can be partitioned into three general classes. If the distribution of *M* is independent of *Y*, the missingness is called “missing completely at random” (MCAR). In this situation, corresponding to the classical model, all missingness can be safely ignored in downstream analysis, with a potential loss in statistical power but no introduction of bias.

If instead the distribution of *M* is dependent on the data matrix *Y*, missingness can be classified in two separate cases. If the dependency can be reduced to simply the *observed* subset of *Y*_*obs*_, where *Y* = *Y*_*obs*_ ∪ *Y*_*mis*_, then the missingness is, somewhat misleadingly, termed “missing at random” (MAR). Data that are MAR cannot be ignored while safely avoiding the introduction of bias. However, due to the restriction on *Y*_*obs*_, the missing values can be probabilistically imputed from *Y*_*obs*_ and added into downstream statistical analysis.

In the worst case, the distribution of *M* is irreducibly dependent on *Y*; such missingness is called “not missing at random” (NMAR). NMAR data cannot be removed without potentially introducing bias, and furthermore cannot be predicted solely from the observed data *Y*_*obs*_. The case of genetic data collection is invariably NMAR, as missingness created by collection technologies such as SNP arrays and sequencers exhibit different performance at different underlying genotypes, and the selection of variants for SNP arrays is itself biased by numerous factors including but not limited to predominant ancestry in early variation projects such as HapMap, variant location in the genome and neighboring sequence content, and allele class at the site of interest.

Genotype imputation is an attempt to project the NMAR missingness of genetic data collection into an MAR condition. Due to linkage disequilibrium (LD), the nonrandom segregation of neighboring variants over a limited number of generations, one can add externally collected, ancestrally related haplotypes to the data (the *Y* matrix). The resulting partition of this matrix *Y*_*obs*_ now ideally contains sufficient information to probabilistically estimate missing genotype data in the original dataset at both typed and untyped variants.

### Multiple Imputation

The framework for Multiple Imputation is established in [9]. Briefly, the method assumes that missing datapoints have been probabilistically estimated using some external method. From these probabilities, an arbitrary *d* complete datasets are drawn from the probability distributions. In the case of standard single-variant analysis, conducting these draws is straightforward as linkage disequilibrium can be ignored.

Each draw is independently subjected to the desired statistical test. This results in *d* sets of $\left\{\beta_{i},s_{i}^{2}\right\}$ effect and standard at each variant. The Multiple Imputation consensus test statistic is computed from the following values:

$$\begin{array}{lll} \beta_{MI} & = & \frac{1}{d}\sum_{i=1}^{d}\beta_{i}\\ s_{W}^{2} & = & \frac{1}{d}\sum_{i=1}^{d}s_{i}\\ s_{B}^{2} & = & \frac{1}{d-1}\sum_{i=1}^{d}{\left(\beta_{i}-\beta_{MI}\right)}^{2}\\ s_{M\;I}^{2} & = & S_{W}^{2}+\left(1+\frac{1}{d}\right)s_{B}^{2} \end{array}$$

Here, *β*_*MI*_ is the consensus effect estimate; $s_{W}^{2}$ is the within-draw sample variance; $s_{B}^{2}$ is the between-draw sample variance; and $s_{MI}^{2}$ is the total sample variance. The test statistic is the ratio of the test statistic and sample standard error, $\frac{\beta_{MI}}{\sqrt{s_{MI}^{2}}}$, which is distributed as T with $\left(d-1\right){\left(1+\frac{ds_{W}^{2}}{\left(d+1\right)s_{B}^{2}}\right)}^{2}$ degrees of freedom. The resulting probability may be interpreted as a posterior probability incorporating both the evidence for association in the study and the actual reliability of the genetic data.

Software implementing this Multiple Imputation regime (in beta) may be found at https://github.com/cpalmer718/statgen-mi. This package features modularized, extensible interfacing with existing analysis software, and bsub/qsub integration. In total, this implementation of MI requires *d* times as long to run, and *d* times as much disk space, as a single analysis run, though running in tranches per chromosome on a cluster can reduce the maximum memory and effective time use by removal of intermediate files and quasiparallelization.

### Meta-Analysis of Multiple Imputation

With Multiple Imputation, a simple regime for seamlessly correcting for different proportions of uncertainty in the contributing analyses is available. Extending the logic used when combining *d* individual MI draws, the following meta-analysis regime applies [22], for M contributing cohorts to a multisite meta-analysis:

$$\begin{array}{lll} \hat{\beta } & = & \frac{1}{M}\sum_{i=1}^{M}\beta_{MI,i}\\ {\hat{s}}_{W}^{2} & = & \frac{1}{M}\sum_{i=1}^{M}s_{MI,W}^{2}\\ {\hat{s}}_{B}^{2} & = & \frac{1}{M}\sum_{i=1}^{M}s_{MI,B}^{2}\\ {\hat{s}}_{meta}^{2} & = & \frac{1}{M-1}\sum_{i=i}^{M}{\left(\beta_{MI,i}-\hat{\beta }\right)}^{2} \end{array}$$

The new variance component ${\hat{s}}_{\text{meta}}^{2}$ is the between-site variance of estimated test statistics. Assuming a balanced study design in which each site runs the same number of MI rounds, the total variance of this *nested model multiple imputation* is ${\hat{s}}^{2}={\hat{s}}_{W}^{2}+(1+\frac{1}{d}){\hat{s}}_{B}^{2}+(1-\frac{1}{M}){\hat{s}}_{\text{meta}}^{2}$. The ratio of $\hat{\beta }$ to $\sqrt{{\hat{s}}^{2}}$ is distributed approximately T with degrees of freedom $\frac{1}{M\left(d-1\right)}{\left(\frac{\left(1-\frac{1}{d}\right){\hat{s}}_{W}^{2}}{{\hat{s}}^{2}}\right)}^{2}+\frac{1}{M-1}{\left(\frac{\left(1+\frac{1}{M}\right)s_{meta}^{2}}{{\hat{s}}^{2}}\right)}^{2}$. In the case of imbalances in the number of draws conducted in each contributing cohort, more complex expressions might be derived, or alternatively a conservative estimate of min(*d*_*m*_) may be used for the weighting factor in the total variance and degrees of freedom.

## Supporting Information

S1 FigRelationship between quality of estimated allelic dosage from minimac3 imputation and predictors of imputation quality.Data are estimated from 10% of the original chip masked from imputation. Discordance of predicted allelic dosage (y-axis) is the fraction difference between dosage computed from imputation probabilities and dosage based on masked genotype data: for example, if the true genotype is reference homozygote and the allelic dosage from imputation is 1.4, the discordance is $\frac{\left|2-1.4\right|}{2}=0.3$. Left panel: imputation quality greater than 0.9; right panel: quality between 0.8 and 0.9. Clusters correspond to minor allele frequencies of 10%; individual bars represent quality stratified by masked genotype. Error bars represent 95% confidence intervals of mean discordance estimate.(TIFF)

S2 Fig Distribution of imputation quality from IMPUTE2 imputation. x-axis: IMPUTE2 info (quality) metric; y-axis: proportion of full set of variants within this quality bin. Distribution is left-truncated at common quality threshold.
(TIFF)

S3 Fig Distribution of imputation quality from minimac3 imputation. x-axis: minimac3 *r*^2^ metric; y-axis: proportion of full set of variants within this quality bin. Distribution is left-truncated at common quality threshold. Final bin with quality greater than 1 indicates small percentage of variants where empirical variance exceeds that of the expected binomial distribution.
(TIFF)

S4 Fig Relationship between quality of best guess genotypes from IMPUTE2 imputation and predictors of imputation quality. Data are estimated from 10% of the original chip masked from imputation. Discordance of predicted genotypes (y axis) is the fraction of best guess genotypes for a given bin that do not match the corresponding masked genotype. Left panel: imputation quality greater than 0.9; right panel: quality between 0.8 and 0.9. Clusters correspond to minor allele frequencies of 10%; individual bars represent quality stratified by masked genotype. Error bars represent 95% confidence intervals of mean discordance estimate.
(TIFF)

S5 Fig Evaluation of the consistency of probability scores from IMPUTE2 imputation, stratified by allele frequency instead of imputation quality. Data are estimated from 10% of the original chip (59808 SNPs) masked from imputation. x-axis: 0.02-width bins of imputation probabilities; y-axis: mean deviation between expected and observed accuracy. Data series correspond to results stratified by genotype class. Left panel: minor allele frequency greater than 0.4; right panel: minor allele frequency less than 0.1. Error bars represent 95% confidence intervals around mean consistency estimate.
(TIFF)

S6 Fig Evaluation of the effect of Multiple Imputation draw count on estimated regression coefficients. x-axis: number of MI draws; y-axis: observed Pearson correlation coefficient between regression coefficient estimates from masked data and estimates from MI on imputed estimates over masked sites. Horizontal line corresponds to correlation between masked data and “best guess” genotypes using imputation probabilities.
(TIFF)

S7 Fig Evaluation of the effect of Multiple Imputation draw count on estimated between-test variance component. x-axis: number of MI draws; y-axis absolute change in between-draw variance component from one MI round to the next. Note this is a traditional boxplot but the boxes are tightly clustered around y = 0, leading to the boxes rendering as the small thick back lines for each x value at y = 0. The emphasis of these results is on the distribution of outliers, corresponding to low-quality imputed variants with trait-correlated uncertainty.
(TIFF)
