# Supplementary figures and images for: Bias Characterization in Probabilistic Genotype Data and Improved Signal Detection with Multiple Imputation
Source: PLoS Genet. 2016 Jun 16;12(6):e1006091. doi: 10.1371/journal.pgen.1006091 (PMC4910998; doi:10.1371/journal.pgen.1006091)

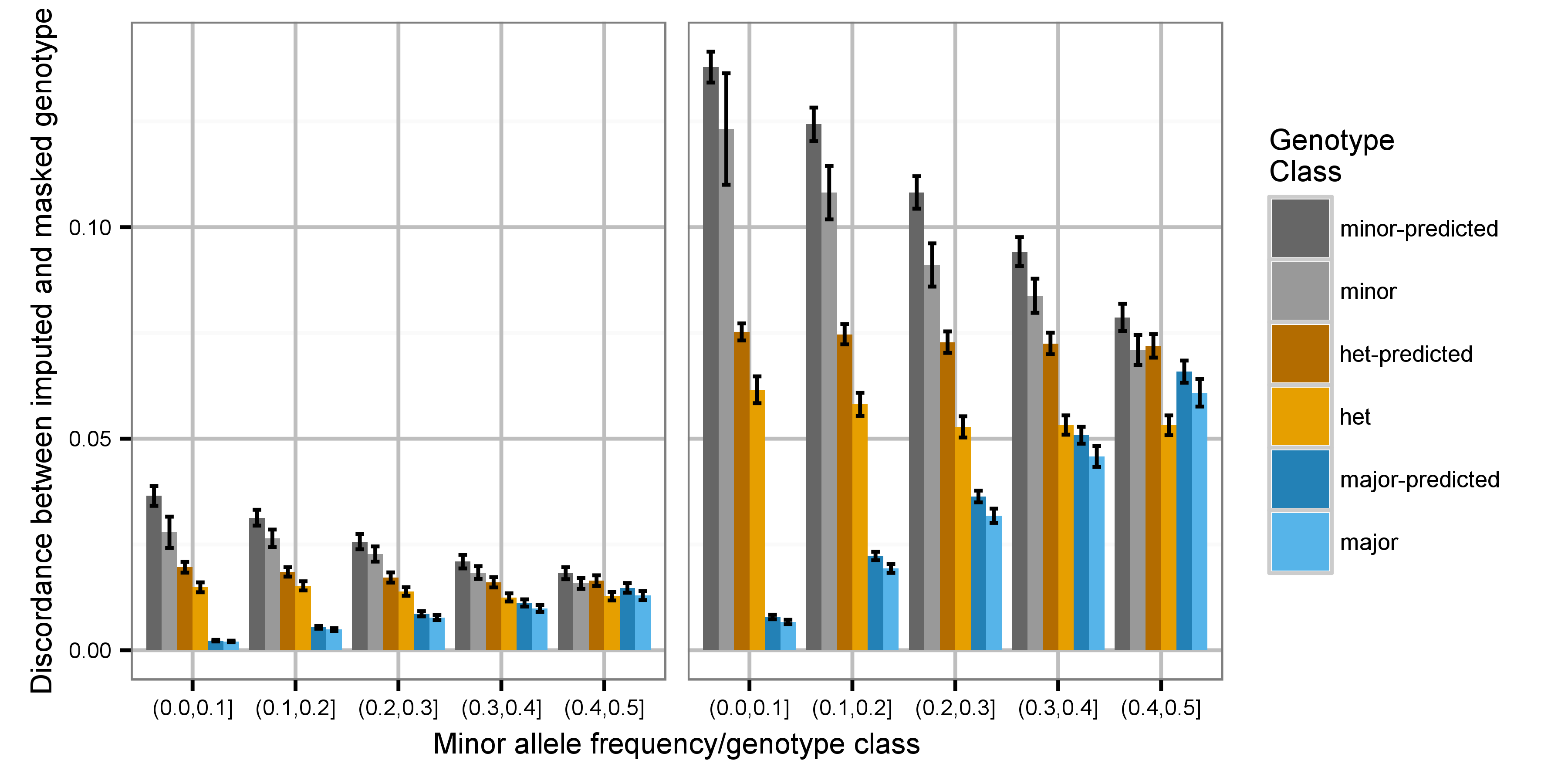

Supplement: S1 Fig — Data are estimated from 10% of the original chip masked from imputation. Discordance of predicted allelic dosage (y-axis) is the fraction difference between dosage computed from imputation probabilities and dosage based on masked genotype data: for example, if the true genotype is reference homozygote and the allelic dosage from imputation is 1.4, the discordance is |2-1.4|2=0.3. Left panel: imputation quality greater than 0.9; right panel: quality between 0.8 and 0.9. Clusters correspond to minor allele frequencies of 10%; individual bars represent quality stratified by masked genotype. Error bars represent 95% confidence intervals of mean discordance estimate. (TIFF) [file pgen.1006091.s001.tiff]

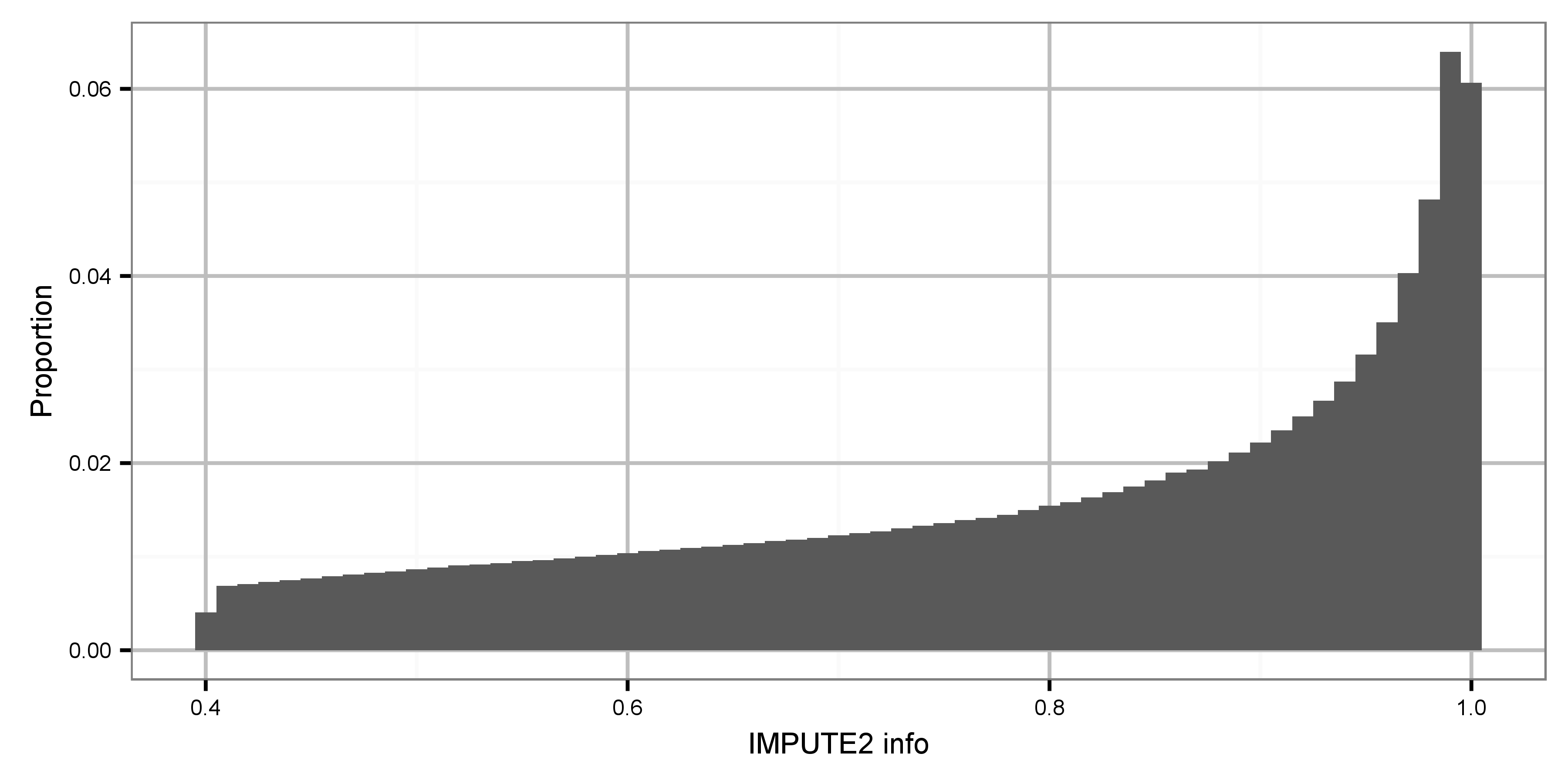

Supplement: S2 Fig — x-axis: IMPUTE2 info (quality) metric; y-axis: proportion of full set of variants within this quality bin. Distribution is left-truncated at common quality threshold. (TIFF) [file pgen.1006091.s002.tiff]

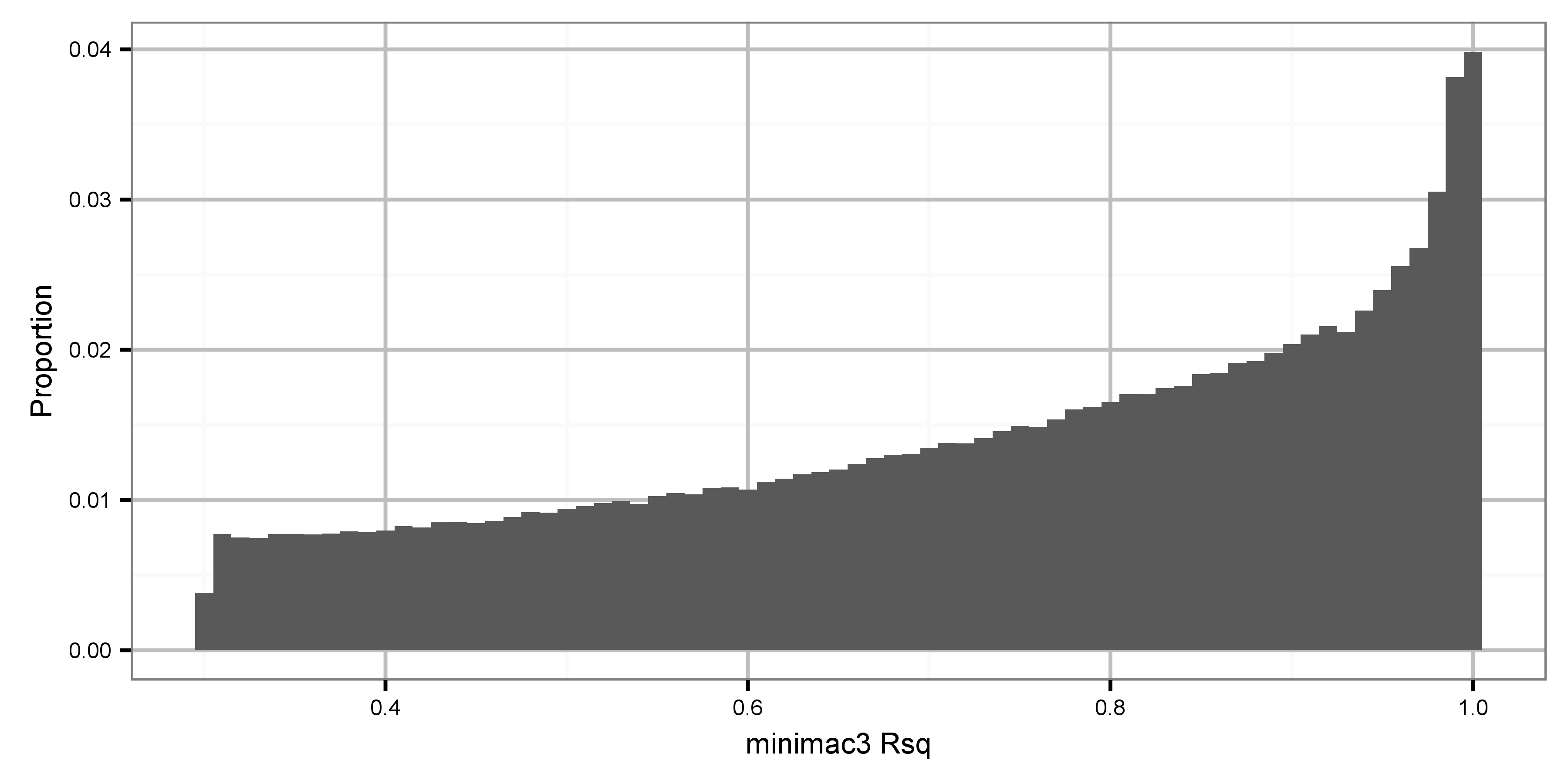

Supplement: S3 Fig — x-axis: minimac3 r2 metric; y-axis: proportion of full set of variants within this quality bin. Distribution is left-truncated at common quality threshold. Final bin with quality greater than 1 indicates small percentage of variants where empirical variance exceeds that of the expected binomial distribution. (TIFF) [file pgen.1006091.s003.tiff]

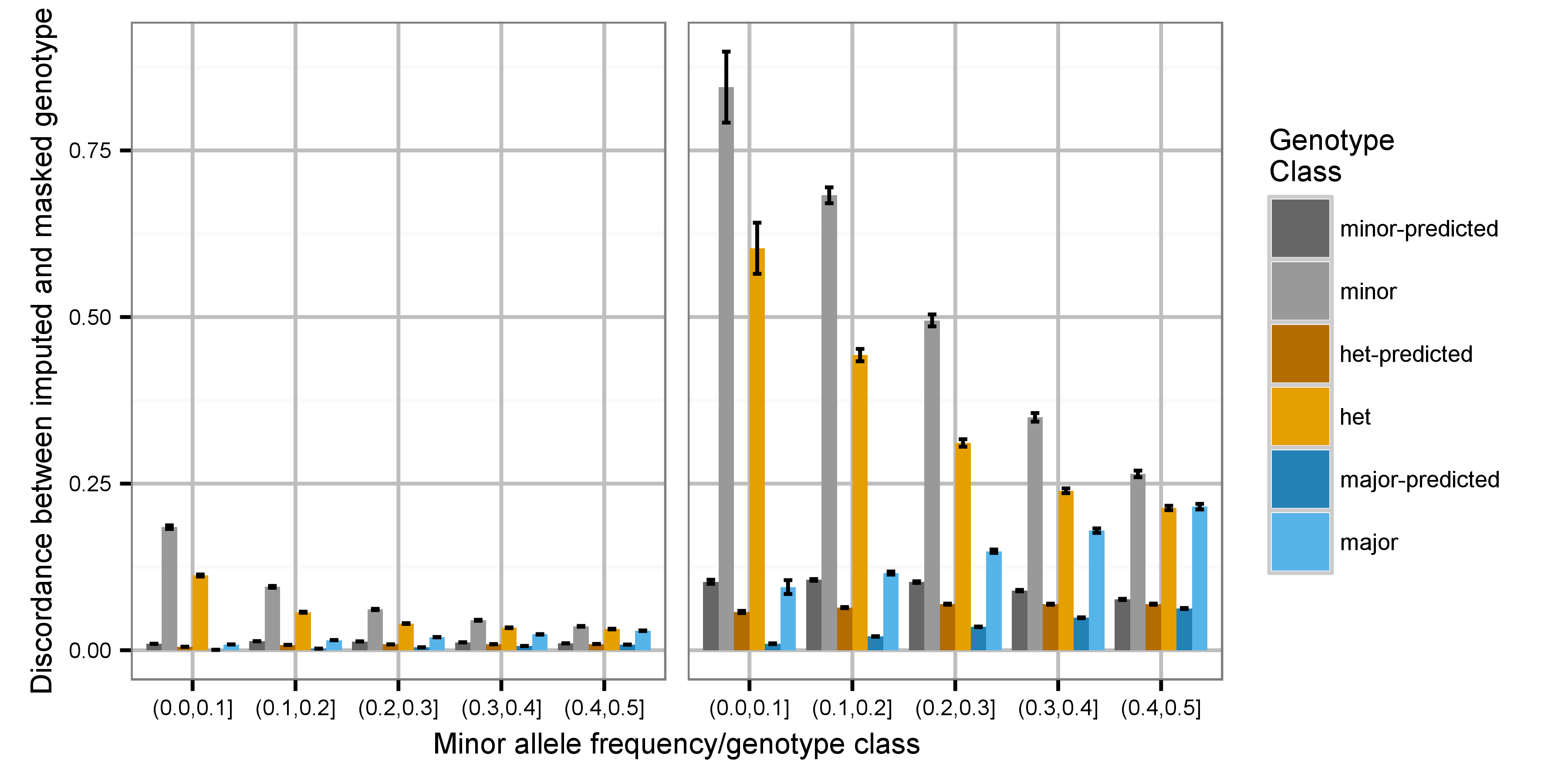

Supplement: S4 Fig — Data are estimated from 10% of the original chip masked from imputation. Discordance of predicted genotypes (y axis) is the fraction of best guess genotypes for a given bin that do not match the corresponding masked genotype. Left panel: imputation quality greater than 0.9; right panel: quality between 0.8 and 0.9. Clusters correspond to minor allele frequencies of 10%; individual bars represent quality stratified by masked genotype. Error bars represent 95% confidence intervals of mean discordance estimate. (TIFF) [file pgen.1006091.s004.tiff]

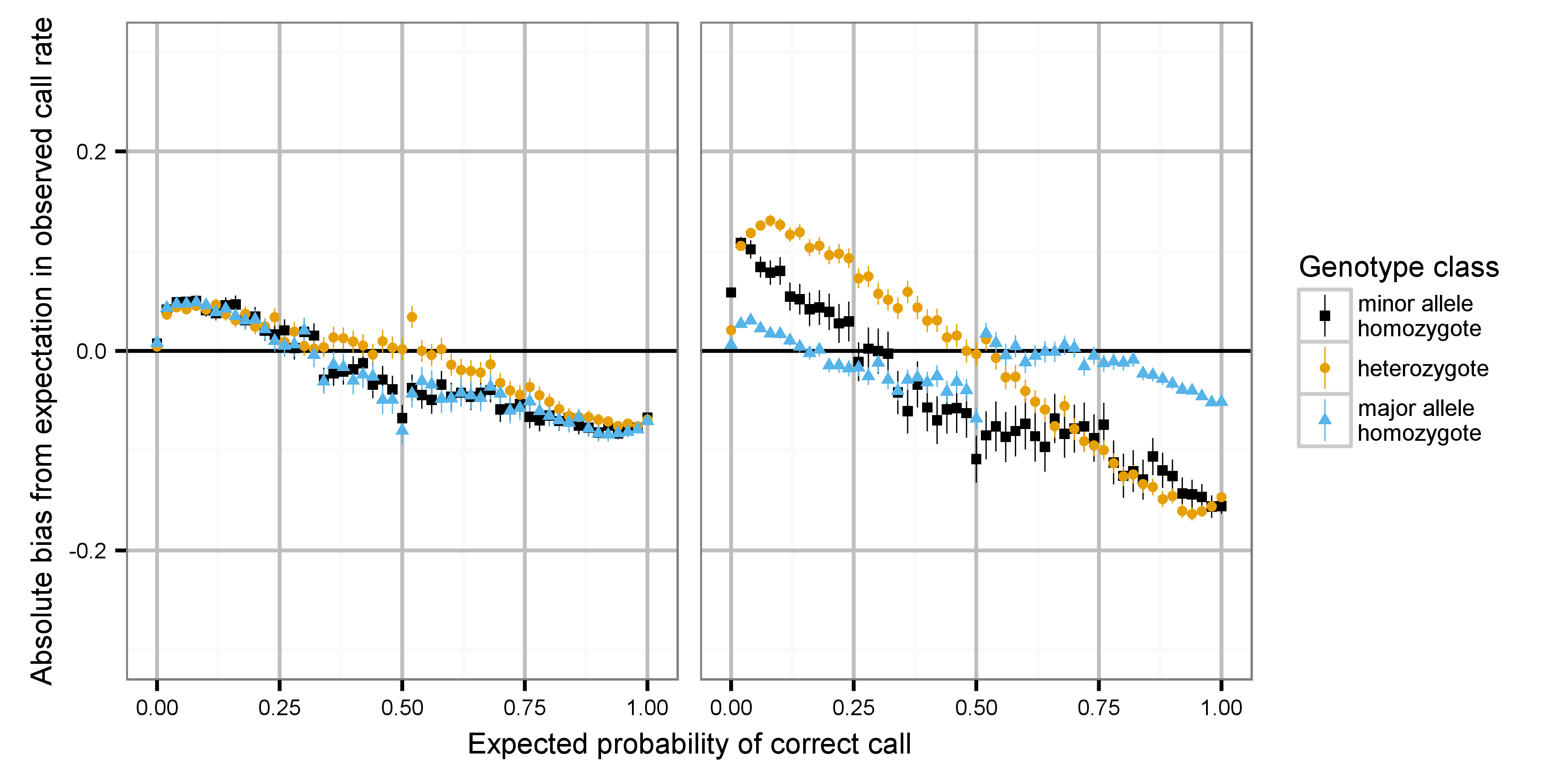

Supplement: S5 Fig — Data are estimated from 10% of the original chip (59808 SNPs) masked from imputation. x-axis: 0.02-width bins of imputation probabilities; y-axis: mean deviation between expected and observed accuracy. Data series correspond to results stratified by genotype class. Left panel: minor allele frequency greater than 0.4; right panel: minor allele frequency less than 0.1. Error bars represent 95% confidence intervals around mean consistency estimate. (TIFF) [file pgen.1006091.s005.tiff]

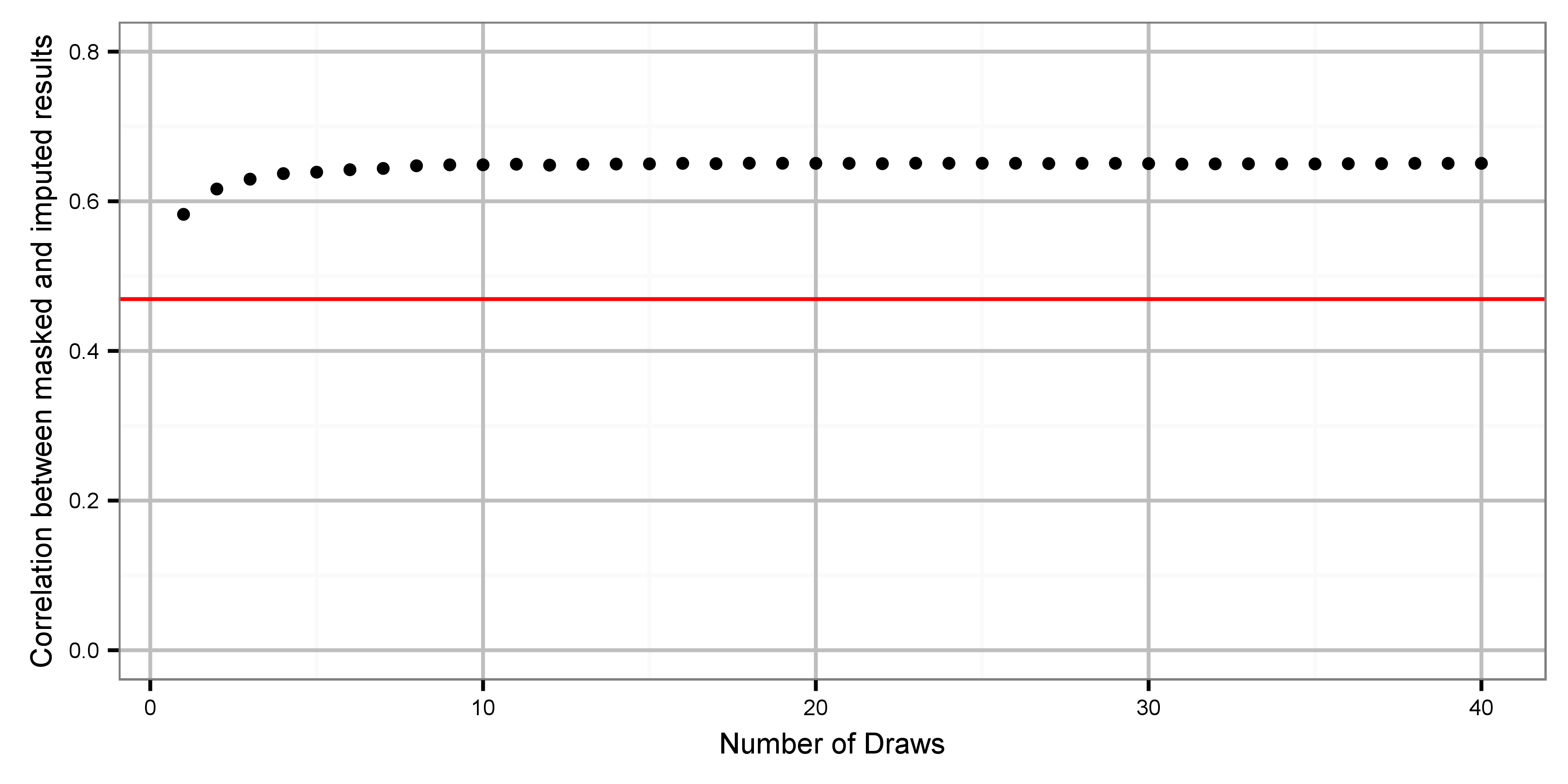

Supplement: S6 Fig — x-axis: number of MI draws; y-axis: observed Pearson correlation coefficient between regression coefficient estimates from masked data and estimates from MI on imputed estimates over masked sites. Horizontal line corresponds to correlation between masked data and “best guess” genotypes using imputation probabilities. (TIFF) [file pgen.1006091.s006.tiff]

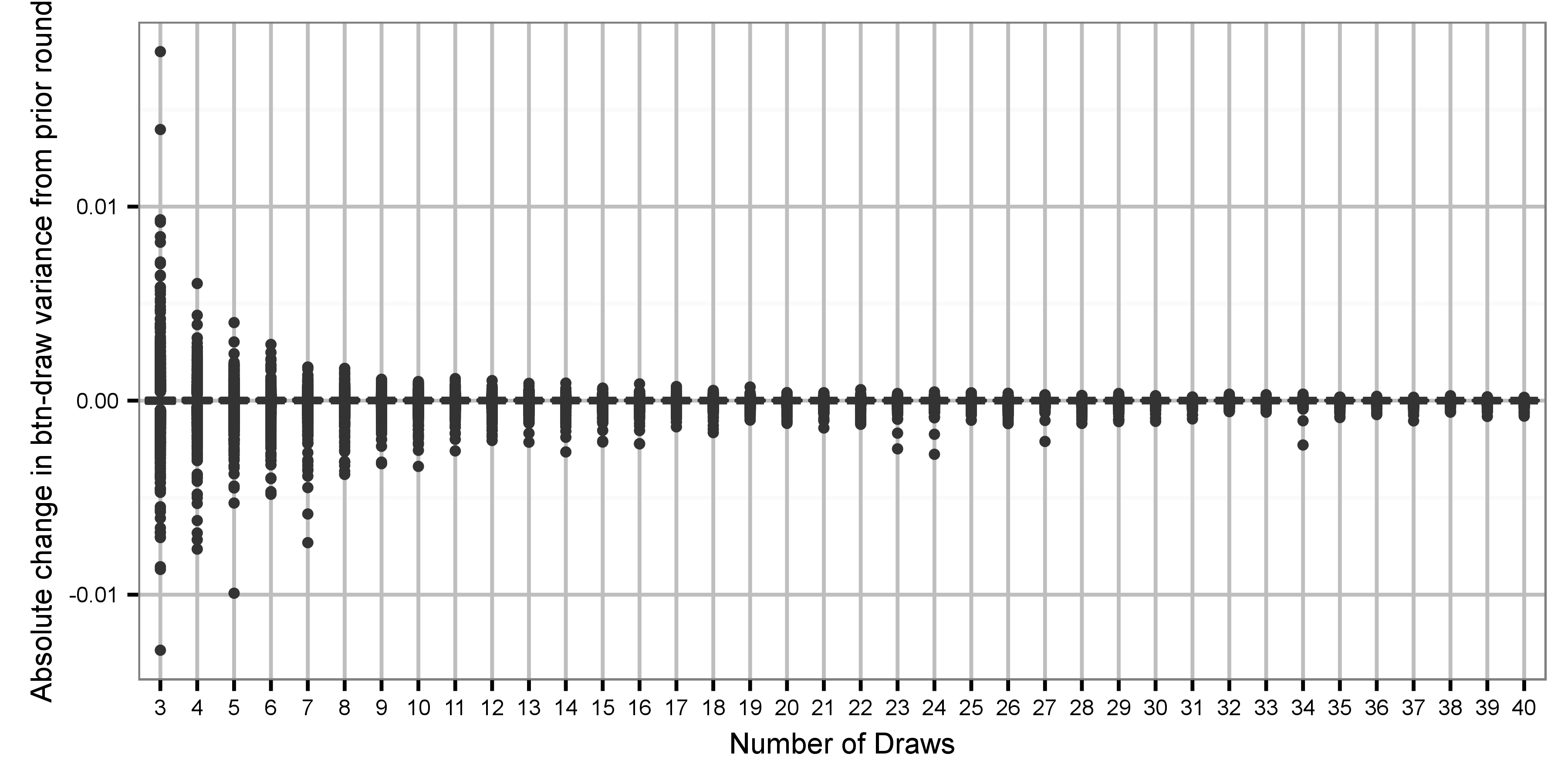

Supplement: S7 Fig — x-axis: number of MI draws; y-axis absolute change in between-draw variance component from one MI round to the next. Note this is a traditional boxplot but the boxes are tightly clustered around y = 0, leading to the boxes rendering as the small thick back lines for each x value at y = 0. The emphasis of these results is on the distribution of outliers, corresponding to low-quality imputed variants with trait-correlated uncertainty. (TIFF) [file pgen.1006091.s007.tiff]
